# Taking the Long Way Around: Towards A Depathologized Ethical Framework of Gender-Affirming Care for Trans Youth

**DOI:** 10.1017/jme.2024.7

**Published:** 2023

**Authors:** Navin Kariyawasam, Nanky Rai

**Affiliations:** 1: UNIVERSITY OF TORONTO, TORONTO, ON, CANADA.

**Keywords:** Gender Affirming Care, Trans Youth, Pathologization, Informed Consent, Medical Colonialism

## Abstract

Political debate regarding trans youth’s access to gender-affirming care (GAC) has pushed many to advocate for GAC by pointing to tragic, pathological outcomes of non-treatment, namely suicide. However, these pathologized arguments are a harmful ethical “shortcut” which should be replaced by a meaningful engagement with the ethics of providing GAC to youth.

In the past year, numerous jurisdictions have placed significant restrictions on youth access to gender-affirming care (GAC), such as puberty blockade and gender-affirming hormone therapies. In the now-overturned *Bell v. Tavistock* decision, the High Court in London ruled that “children are highly unlikely to be able to consent to taking puberty blockers.”^1^ As of July 2023, twenty U.S. states have passed laws or policy banning gender-affirming care.^2^ These restrictions parallel those in the U.K. as broadly based in the stance that young people cannot consent to GAC. In 2022, Texas Governor Greg Abbott directed the Texas Department of Family and Protective Services to classify the provision of GAC to youth as child abuse, and to investigate families that enable such care.^3^ This spate of legislation is unequivocally harmful to trans youth.^4^ The authors of this paper endorse fulsome youth access to GAC. Further, these developments reveal a more profound drive to maintain domination through the enforcement of cisheteropatriarchy as an essential feature of settler colonialism. Regrettably, the mainstream response from trans-supportive clinicians has, for the most part, not centered on a liberatory approach to the rise in regressive transphobic policies, but rather reinforces a problematic rhetoric of illness and tragedy rooted in saviourism to justify youth access to GAC.^5^

Advocates, both clinicians and caregivers, have highlighted the supposed tragic outcomes of denying GAC to youth, such as suicidality, self-harm, and depression. These treatments are often described as “lifesaving,” and their legislative bans are said to “deny life.”^6^ Clinician responses to bans on care often centre on suicidality, and liken gender-affirming care to other lifesaving treatment such as antibiotics for a bacterial infection.^7^ A paper detailing parent and caregiver perspectives on the legislation discussed above is provocatively titled, “This Could Mean Death for my Child.”^8^ Clinicians at the Pediatric and Adolescent Gender Clinic at Stanford Children’s Health describe the denial of care as “psychologically devastating,” and even ethicists seeking to justify GAC for youth will often point to the miserable outcomes of treatment omission as a core argument in support of access.^9^ In a passionate speech to the Iowa state senate regarding a proposed ban on GAC, State Senator Zach Wahls proclaimed “…kids are going to kill themselves because of this law. Iowa children will die if this becomes law. That’s what will happen.”^10^ The impetus to rescue trans youth from the suffering they would endure with non-treatment is thus presented as the primary ethical justification for such care.

In so doing, clinicians reinforce rather than problematize the social and political forces aimed at restricting the right to autonomy for trans youth. Sahar Sadjadi identifies this concerning trend, critiquing dominant narratives of saviourism and a “looming disaster of puberty.”^11^ Sadjadi argues that this sensationalism is a problematic tactic, not only in that it locates pathology within the individual trans child, but also in that it obscures a meaningful discussion of the ethics of GAC for youth. Opponents of youth access to GAC have recognized this rhetorical avoidance, calling it the “suicide card,” and pointing out the ways in which such rhetoric appears to flee from a meaningful discussion of youth autonomy and capacity to make these medical decisions.^12^

In this paper, we will review critiques of the pathological framework of GAC provision to youth, drawing upon work both in the clinical setting as well as by theorists and scholars outside of medicine. We will then briefly review theoretical and applied depathologized frameworks that more responsibly engage with and honour the autonomy of trans youth.

To be sure, the narratives of suicide and pathology used to defend youth access to GAC are well-intentioned. They are also often accurate. Youth are likely to experience worse mental health outcomes as a result of restricted access to GAC, and we do not seek to deny the realities of such transphobia. However, these inherently pathologized arguments are ultimately a harmful ethical ‘shortcut’ which should be replaced by a liberatory praxis of healthcare for trans youth.

We argue that the pathologization of gender diversity exists within the framework of settler colonial violence and therefore reinforces rather than challenges the rise in regressive policies attempting to erase the very existence of gender variance. The amplified and reinforcing nature of patriarchy, heterosexism, and transphobia/cisnormativity — aptly defined as cisheteropatriarchy^13^ — cannot be understood outside of the context of colonialism, in particular settler colonialism. Patrick Wolfe characterizes settler colonialism through the “logic of elimination,” whereby eliminating colonized peoples allows for the theft of Indigenous territories and the foundation of settler society.^14^ In addition to the logic of elimination, Luana Ross describes colonialism as a logic of incarceration where Indigenous people are “confined in forts, boarding schools, orphanages, jails and prisons and on reservations.”^15^ As part of the logics of elimination and incarceration, mechanisms of control and domination are created through the enforcement of White supremacist and Eurocentric social norms.^16^ As described by Lugones’ framework of the ‘coloniality of gender,’ the process of colonial settlement required the enforcement of normative gender in order to construct relationships rooted in power and domination.^17^ This construction afforded European colonizers, particularly settlers, a sense of superiority which justifies the attempted genocide of Indigenous peoples and the ongoing invasion, theft, and occupation of Indigenous territories.^18^ Indigenous scholars Arvin et al. remind us of the political, intellectual, and ethical imperative of identifying the ongoing process of settler colonialism as intertwined with that of cisheteropatriarchy, where the enforcement of Euro-Christian frameworks of the gender binary is also used as a tool to delineate ‘legitimate’ citizenship and access to land.^19^ The maintenance of settler colonial domination relies on the ongoing imposition of social hierarchies rooted in White supremacy, capitalism, and cisheteropatriarchy.^20^ This allows for the ongoing unjust production of the *superior* (read: socio-bio-psychologically *normal*) therefore *deserving* settlers who can maintain access to occupied lands and resources. While this paper focuses on the need to dismantle rather than enforce the pathologization of gender variance within clinical care, our critique must remain grounded within the broader context in which pathologization is used as a mechanism of control to maintain settler colonial domination.

In this paper, we will review critiques of the pathological framework of GAC provision to youth, drawing upon work both in the clinical setting as well as by theorists and scholars outside of medicine. We will then briefly review theoretical and applied depathologized frameworks that more responsibly engage with and honour the autonomy of trans youth. Our aim is that clinicians who work with trans youth begin to rethink the ways in which they interact with patients and advocate for their access to GAC in the face of contemporary attacks on their autonomy. Ultimately, the authors argue that the pathologization of gender diverse people, including trans youth, is an integral aspect of settler colonial assimilation processes. Deconstructing the dominant modes of thinking around trans youth and their supposed need for treatment must be understood in the context of a broader anti-colonial practice that seeks to name, resist, and dismantle settler colonialism, cisheteropatriarchy and white supremacy.

## Critiques of the Pathological Framework

The justification of youth access to GAC through narratives of tragedy and suicide is undeniably imbricated within a broader positioning of trans bodies as inherently sick and in need of correcting by medicine. Heyes and Latham identify this, arguing that such medical narratives construct suffering as being constitutive of transness.^21^ This pathologized ethics of GAC for youth can be understood through Eve Tuck’s analytical lens of “damage-centred research.”^22^ Tuck identifies that while such research is often used to leverage reparations or other progressive ends, it is ultimately “a pathologizing approach in which the oppression singularly defines a community.”^23^ Tuck draws linkages between damage-centred research and settler colonialism, where damage-centred research is instrumentalized to bolster eugenic projects rooted in white supremacy and global capitalism, allowing for the ongoing domination and exploitation of Indigenous peoples and other dispossessed communities. As such, medical institutions’ reliance on damage-centered approaches to trans youth continues to reinforce supremacist ideologies based in settler colonialism. Tuck presents desire-centred research frameworks as a way forward. These frameworks centre the full subjectivity of people involved and are equipped to highlight structural inequity.^24^ This will be explored in further detail in the second part of this paper.

In light of the critiques reviewed here, activists have been calling for depathologization in trans medicine. The International Campaign Stop Trans Pathologization is a platform that denounces the effects of pathologization, such as the removal of autonomy for trans patients and the imposition of restrictive and invasive evaluations.^25^ This discussion has grown to include trans youth as well, with proponents of depathologization noting higher risks of discrimination, coercive treatment, and binary conceptions of gender under a pathologized model.^26^ However, as much of the literature cited above indicates, this position is still an emerging one, with the dominant position remaining pathological with respect to trans youth.^27^

The dominance of the pathologized model is clearly seen not only in the ethics and advocacy cited above, but also in the diagnostic criteria required for youth to access GAC in the first place. The definition of gender dysphoria in the *Diagnostic and Statistical Manual of Mental Disorders* (DSM) requires a finding of “clinically significant distress” for at least six months.^28^ Guidelines also continue to recommend a diagnosis of dysphoria before GAC is initiated.^29^ As such, clinicians expect to see trans youth in distinctly distressful and pathologized states in order for them to access GAC. The most recent World Professional Association for Transgender Health (WPATH) Standards of Care 8 recommend that youth meet criteria for an ICD-11 diagnosis of gender incongruence, which represents some progress as it does not directly name distress or pathology, but still requires a marked and persistent incongruence for diagnosis.^30^ Diagnostic manuals and clinical guidelines^31^ also shape the views of policymakers and the general public, defining conceptions of normalcy and psychopathology.^32^

Pathological frameworks in the justification of GAC for youth, while hegemonic in medicine and even mainstream trans advocacy, ultimately disempower trans youth and limit their future life options. As such, we explore both the individual and institutional level impacts of a pathologized approach to GAC for trans youth below. Only then, can we better understand the ethical imperative to move beyond this approach.

### Impacts of a Pathologized Approach

#### Individual-Level Impacts

Pathological approaches to the ethics of GAC for youth have a number of harmful effects on the person, the most obvious of which is the label of pathology itself. Such a label is not only inaccurate, in that gender diversity is not pathological, but also functions to attribute any discomfort or distress that trans youth may experience as inherent to an illness within them, as opposed to the structures of the society in which they live.^33^ This is not only problematic in and of itself but also, as Horowicz argues, limits therapeutic approaches, in that diagnostic criteria become individual symptoms to treat, limiting a holistic approach to the youth’s needs.^34^

Proponents of the pathological model may defend the current framework in that distress and dysphoria are not posited as inherent to trans identity, but rather a result of living within a transphobic society. Such an argument is disingenuous, given the frequent requirement of distress for access to GAC, especially in the case of youth, whose advocates rely heavily on justifications based in suicide prevention and mental illness. Indeed, many advocates will simultaneously acknowledge that dysphoria is not constitutive of transness, yet present the necessity of GAC as based in its role as a treatment for dysphoria.^35^ Clearly, pathology and distress are seen as core to trans identity, as they are often the only path to accessing GAC, both ethically and clinically.

In addition to the pathological label itself, pathologization also surreptitiously reifies a binary conception of gender in both medicine and trans youth themselves. The pathologization of gender variance is historically and discursively based in attempts to preserve a gender binary.^36^ Such a binary schema of gender in medicine was deliberately constructed so as to quell fears of a third sex and other such non-binary bodies.^37^ Nelson writes:

That is, society could rest easy with medicine pathologizing gender ‘deviance’ and proposing a clinical strategy for explaining and containing it: nobody’s genitals were going under the knife unless they had the right kind of illness, and besides, nothing that happened in an operating room on any single patient could really challenge gender’s ‘fundamental truths’ — e.g., that there are two and only two, that everyone has one or the other, and which one you are is determined by some deep and immutable fact.^38^

This binary continues to be starkly visible in DSM criteria.^39^ With minimal acknowledgement of non-binary identities, the breadth of diagnostic criteria focus on a desire to be the “other gender.”^40^ This is even more apparent for children, with criteria focused on masculine and feminine toys and games, “cross-dressing,” and again the desire to be the “other gender.”^41^ The same normative beliefs around trans youth are seen in the previous WPATH Standards of Care 7, whose recommendations continue to influence countless guidelines worldwide.^42^ A qualitative report on trans youth experiences at the Gender Diversity Clinic in the Children’s Hospital of Eastern Ontario (CHEO), by SAEFTY Ottawa, found that patients were often uncomfortable with these questions about toys and clothing, feeling that “they perpetuated cissexist and binary understandings of gender.”^43^ Similar experiences were reported in New Zealand, where young adults felt that readiness assessments were designed to establish whether they were “trans enough.”^44^

Critically, the binary framework of gender in medicine, rooted in pathology, is not only inaccurate, but plays a critical role in the reproduction of the gender binary within clinical environments and society at large. Through the construction of difference as pathology, the maintenance of a normative gender model has been used to regulate and eliminate “deviant bodies.” This construction has allowed for the preservation and securitization of settler colonial nation states and global capitalism, through the elimination of deviance and supremacy of the “normal” elite.^45^ Healthcare professionals, as gatekeepers to GAC, are positioned as adjudicators of authentic gender, ultimately producing the gender that they ostensibly observe and diagnose.^46^ This is particularly pernicious in the early stages of trans identity development, where young people often feel they must choose a path so as to access GAC, for which treatment protocols are also highly binarized.^47^ When clinical practice regarding gender is formulated in a binary, youth are often coerced into conformity, in order to be legible to the clinicians who dictate their access to care.

This coercion not only happens at a sociopsychological level, but at a somatic one as well. SAEFTY’s report found that youth also felt pressured to follow a typical “cookie-cutter” path in their medical transition, even when this did not reflect their own transition goals.^48^ This approach, which always begins with puberty blockade and ends with surgery to reflect a binary gender, has been identified and problematized thoroughly.^49^ The prevalence of youth seeking ‘partial’ treatment (treatment that does not follow a binary path) is increasing, yet they are often denied care because they do not meet the full criteria for diagnosis.^50^ In this way, diagnostic criteria erase the individual subjectivity and gender constellation of trans youth, particularly those who are non-binary. Konnelly identifies this transmedicalist framework as one which pressures non-binary people to push themselves into a binary in order to be legible to providers.^51^ This ideology, for which the medical institution is responsible, even begins to seep into one’s own experience of their identity,^52^ something to which youth are especially vulnerable.

The binary framework of gender resulting from a pathologized and diagnostic model also impacts the therapeutic relationship trans youth have with their clinicians. When youth are aware that they must meet certain diagnostic criteria in order to access GAC, they will often overemphasize the elements of their experience that fit normative medical models: performing a gender that their clinicians will deem legible and worthy of treatment.^53^ This is observed empirically, with youth in SAEFTY’s report similarly describing a pressure to perform stereotypical gender, and at times lying to clinicians so as to access treatment.^54^

That youth may be required to perform a false gender experience to access GAC must primarily be read as a form of medical gender regulation. Dean Spade argues that such requirements constitute medical governance, designed to regulate trans folks and reify normative gender.^55^ This is, again, used as a discursive tool to retain regulatory and colonial power over bodies labeled as deviant. There are also harmful clinical implications. Trans youth often do benefit from therapeutic support in the exploration of their gender, especially if these supports do not exist elsewhere. However, the clinicians who care for them play a dual role of gatekeeper and support. These two roles are in conflict, where youth benefit from genuine honesty and exploration of their uncertainties, while simultaneously aware that they must perform a level of normativity and assuredness so as to retain access to care.^56^ The limits therefore placed on trans youth’s ability to disclose to their clinicians are a clear harm to their overall care. Furthermore, Ashley calls for us to embrace an “ethics of exploration,” rather than seeking to identify and predict a stable gender concept in young people.^57^ This is explored further below.

The pathological model places further barriers to access on trans youth. For instance, to satisfy diagnostic criteria, youth are often asked deeply invasive questions. They may be questioned on their relationship to their genitals, the toys they played with as children, or their family dynamics. Young people report feeling uncomfortable with the invasiveness of such inquiry, expressing that it often feels as though it is to satisfy a clinician’s curiosity.^58^ Yet invasive questions are inherent to a model that must locate a pathology within the body in order to justify care.

It is critical to recognize the basis of pathology in the patient impacts detailed above. Requiring a pathological diagnosis for access to GAC invites the rigid diagnostic criteria that constrain gender to a binary, restrict treatment protocols, and require performance and invasive evaluation. When ethicists and clinicians advocate for youth to access GAC on the grounds that they will suffer tragic outcomes if left untreated, such narratives uphold a medical discourse that places trans youth as inherently ill, while evading the true ethical questions at play.

#### Institutional Impacts

As stated in the introduction, pathologized narratives of trans folks must be understood contextually within a broader system of settler colonialism through “the imposition of the settlers’ gender and sexuality systems of cisheteropatriarchy.”^59^ The diagnostic model fixes gender into a stable concept, inherent to the body it inhabits. Trans theorists have critiqued the medical approach to trans children in that it is formulated to taxonomize and order gender.^60^ This is intimately tied to a pathological model, where GAC is constructed as a remedial treatment to a classified disease.^61^ Such a construction not only limits the gender possibilities of young trans patients, but also plays a significant role in a broader system of gender regulation.

The taxonomical and classificatory framework inherent to the pathological model of trans medicine has been problematized by trans scholars as medical and psychiatric colonization.^62^ This term is important, in that it identifies the profound imbrication of pathologization within systems of power that continue to label, regulate, and oppress bodies considered deviant. Diagnostic manuals and narratives of the tragic, inherently ill trans patient position that person as a pathological, exotic being, in need of study and medical salvation.^63^ A discursive separation is made between the “us” of medical science and “them” of trans communities who seek GAC. It is upon this foundation that narratives of saviourism (intimately tied to notions of supremacy) can be built. The argument that trans youth need access to GAC to save them from their eventual suicide and mental illness plainly cooperates with such a narrative.

The othering of trans youth as a politically distinct “them” is observable in the asymmetric application of ethical protections to trans youth compared to cisgender youth. This asymmetry is highlighted by Milrod, addressing the argument that the irreversibility of GAC makes youth consent to such care impossible.^64^ Milrod identifies that similarly irreversible procedures are regularly offered to cisgender youth in a variety of settings. A more ludicrous example can be seen in Pilgrim and Entwistle’s ethical discussion on youth capacity to consent to GAC, where a single case of necrotizing fasciitis after gender-affirming surgery is cited as a meaningful consideration in the broader ethics of such procedures, as though necrotizing fasciitis is not an equivalent risk in countless procedures performed for young patients.^65^ Trans youth here are surreptitiously othered as politically and ethically distinct from their cisgender peers, furthering a broader regulatory project of labeling and segregating deviance.

Crucially, medicine is deeply involved in this politicization and disproportionate regulation of trans youth. Medicine not only plays a considerable role in trans governance and legal recognition, but also holds almost unilateral control over the determination of viable and non-viable forms of life.^66^ Again, it is pathologization that enables such tight regulation of trans politics, wherein psychiatric dominance and gatekeeping to GAC enable a discursive monopoly on legitimate gendered expressions and ways of life.^67^

Critics of pathologization have established a clear link between pathologizing trans identity and resultant desires to treat and prevent such a “medical condition.”^68^ The most dramatic example of this is the continued legitimacy given to the ethics of preventing trans identity in youth altogether, which is often presented as a reasonable ethical debate.^69^ It is deeply concerning that such eugenic principles are given any credence, even to the point that the WPATH must explicitly identify attempts to prevent trans development as “no longer considered ethical.”^70^ Here again, the connection to settler colonialism must be reiterated. One of the most pernicious weaponizations of medicine within colonialist projects is through eugenics and the “prevention” of deviance. This allows for the maintenance of Eurocentric colonial hierarchical psychosocial processes that afford settlers control over peoples, lands, and resources.^71^ That preventing non-normative identities and expressions continues to be discussed in earnest represents the ongoing impacts of cisheteropatriarchy and White supremacy as key features of colonial ideology that remain embedded within medicine.

Beyond such a dramatic instantiation of pathologizing medical governance, attempts to make the body more normative are also clearly visible in GAC for trans youth. One of the goals of gender-affirming care for pubertal youth is described as enabling “a transgender individual to blend into society more easily as their affirmed gender.”^72^ Gill-Peterson problematizes arguments used in trans youth medicine that early transition enables easier passing and a more normative body.^73^ Such rhetoric posits the value of early transition in its ability to ultimately reduce trans visibility and maintain a visible gender binary. Again, this argument is rooted in pathology, where early intervention enables the treatment of the deviant nature of transness, so as to enable greater assimilation into cisgender society. This is not to invalidate the very legitimate desires that many trans youth may have to ‘pass’ as normatively gendered people, but rather to problematize the broader medico-social society that demands such presentation. As such, Gill-Peterson calls for us to reimagine the clinic entirely, with centrality placed on what trans children say about themselves.^74^

And in light of the plethora of individual and systemic harms of pathologization, this reimagined clinic must be completely devoid of a pathological model. The only way to deconstruct the continued medical coercion and investment in colonial gender systems in GAC for trans youth is to remove the diagnosis entirely: a conclusion already reached by key scholars.^75^ This is not to disregard the work of innumerable trans activists who have used a pathologized framework to achieve recognition from which trans youth benefit today. As Krieg notes, pathological classifications of trans people were formed in a hostile socio-political context, where a conception of trans people as sick was the most socially palatable means of legitimizing treatment.^76^ This approach, while flawed, was meant to bring the community genuine benefit, and those who espoused such models should not be vilified. Rather, we must envision the next chapter of clinical care for trans youth as moving past and through pathologization.

A more responsible approach in the ethics of GAC for trans youth centres their autonomy and their prerogative to self-determination. However, to rid ourselves of a pathologized framework of youth consent to GAC leaves us with an important ethical task. Even scholars critical of a medicalized and pathological approach to trans medicine recognize the need for building clinical care that is rooted in bidirectional accountability, including informed collaborative decision-making and capacity assessments.^77^ How do we offer healthcare to trans youth without positioning them as sick? How do we evaluate their capacity without erasing their autonomy? If we reject the ethical shortcut of pathologization, what ethical pathways exist for providing GAC to youth?

## Taking the Long Way Around

### Rethinking Gender-Affirming Care for Trans Youth

Developed as an alternative to the standard diagnostic model, the informed consent model emphasizes patient autonomy and a collaborative approach towards care.^78^ This model removes requirements of external evaluations and diagnoses, acknowledging that the person is often best positioned to evaluate their benefit from treatment options (with risks and benefits discussed with their healthcare provider). Central to this model are the person’s own experiences, understanding, and leadership in clinical decision-making. Rather than having clinicians diagnose a need for treatment, the person seeking GAC is informed of their options and the associated risks, and a decision is made collaboratively.

Leaders in adult trans medicine have embraced the informed consent model, one of its pioneers being Fenway Health in Boston, Massachusetts, and increasing uptake is being seen now in hundreds of clinics. Empirical research on adult experiences at these clinics is encouraging.^79^ Analogous models are also being legislated in certain jurisdictions, such as in Argentina’s Gender Identity Law,^80^ which allows self-identification with no additional requirements.^81^

While the informed consent model continues to grow in support in adult trans medicine, as Clark and Viriani note, youth are still required to undergo the diagnostic and pathologizing requirements of older models of care.^82^ In opposition to this, Clark and Viriani advocate in favor of an informed consent model for trans youth as well. They present both a deontological and consequentialist imperative for an informed consent model in GAC for youth, as well as their empirical research in a youth gender clinic in British Columbia, Canada.^83^ They found that youth aged fourteen to eighteen were able to demonstrate sufficient understanding of GAC to provide informed consent, and that youth recognized the significance of their decision, distinguishing it from less consequential decisions they might make in their regular life.

Yet, even the informed consent model can be applied with a pathologizing lens, with clinicians still perceiving their care as rectifying an illness. Its heavy reliance on a vague clinical judgement of capacity also enables inconsistency and bias, which is likely to disproportionately affect poor, Indigenous, Black, other racialized, and/or disabled youth.^84^ While a fulsome exploration of racial bias in the provision of GAC to trans youth cannot be done justice presently, it is critical that novel frameworks of care actively engage with the intersectional marginalization of trans youth and account for the ways in which they are likely to face multiple axes of oppression when seeking care.

### Celebration, not Cure

In addition to an informed consent model, a fundamental shift in the provision of GAC to trans youth may proffer a more fulsome understanding of the role GAC plays in their development. Ashley invites us to interrogate the primary assumption in the status quo debate that youth are cis by default.^85^ Instead, Ashley advocates for an “ethics of exploration” rather than an “ethics of prediction.”^86^ While gender exploration is presently seen as a precondition to GAC, exploration can in fact be achieved through it. In simplified terms, this means that we ought not assume that GAC is meant to affirm a previously determined, stable, and static gender, but rather that a fulfilling gender can be explored and built through GAC. Similarly, Kai Cheng Thom calls on us to let go of the illusion that we can be sure of a static and immutable gender and instead embrace the messy complexity of gender and life.^87^ In this approach, fears of desistence make little sense. Furthermore, such thinking stands in contradistinction to the implicit prevention of trans identity throughout 20th century medicine, by enabling the want and desire for trans life to develop and flourish.^88^ In Ehrensaft’s words, we must “learn to live with gender ambiguity and not pressure our children with our own need for gender bedrock.”^89^

Critiques of pathology and tragedy as the impetus for medical care are far from new, and there is a vast body of work from which more responsible models can be gleaned, many of which are already in practice. Developing such models further is out of scope presently, but there is much to be learned from a review of existing literature. In Tuck’s critique of damage-centred research, a desire-centred framework is posited as a possible antidote.^90^ In Tuck’s words, “desire-based research frameworks are concerned with understanding complexity, contradiction, and the self-determination of lived lives.”^91^ Desire, in this case, is formulated as generative, engaged, and centred on the full subjectivity of those involved. Such a framework flips the script of blame and responsibility and is better equipped to expose structural inequity. A desire-centered framework calls for an epistemological shift, where the goal is not to “paint everything as peachy, as fine, as over” and rather “accounts for the loss and despair, but also the hope, the visions, the wisdom of lived lives and communities.”^92^ This depathologizing framework resists domination and the creation of the subhuman, instead moving us all towards upholding people’s right to self-determination, which the authors believe is a necessary prerequisite for health and well-being.

Other work highlights the value of dignity in ethical deliberation regarding GAC in youth.^93^ Here, GAC is not seen as a treatment to an inherent illness nor salvation from ensuing tragedy, but rather as a means by which medicine can support trans youth in accessing dignity and self-actualization. These are meaningful elements of well-being and represent legitimate goals of healthcare in their own right. SAEFTY’s report provides examples of what this could look like clinically. They suggest evaluating young patients by focusing on experiences that bring about gender euphoria, comfort, and joy.^94^ Such an approach clearly focuses more on supporting youth in accessing their well-being, rather than diagnosing a pathology of dysphoria.

A significant body of literature also suggests formulating the interface between trans embodiment and medicine as similar to that of disability medicine. Based on the work of Robert McRuer, Krieg applies the social model of disability to trans medicine.^95^ The social model locates trans issues as contextualized within a gendered and transphobic society, built in contradistinction to a medical model, which locates pathology within the trans body. This model leans heavily on work in crip theory and disability justice, where disability is also located in an ableist and inaccessible society, rather than the individual.^96^ Such a model would enable a more responsible ethics of GAC for trans youth, in that they would be seen as having distinct access needs for which they may seek medical support.

Using a disability justice lens offers two clear benefits. The first is that the disadvantages and possible distress experienced by trans folks are seen as rooted in the society in which they live. As such, GAC would not be seen as correcting a bodily pathology that causes distress, but rather supporting trans youth inhabiting a cisheteropatriarchal world.^97^ The second benefit is that GAC itself would not be seen as the treatment of an illness. This rejects the common narratives in contemporary ethics for this field of care, which rely on the justification of treating a tragic illness, instead identifying unique access needs that trans youth have. This not only removes a stigmatizing and restrictive pathological label, but also fosters a more collaborative approach to GAC in trans youth, where their unique access needs are considered, rather than a “cookie-cutter” treatment path being foisted upon them.

The implications of this paper can expand beyond trans medicine as well. The ethical quandary presented by youth access to GAC offers a more expansive enlightenment on status quo frameworks of pediatric consent.

The use of a disability justice model is not without criticism, with legitimate concerns expressed about the continued reliance on a form of diagnosis, whether a disability or pathology.^98^ The approach will have to be carefully built, but there is certainly much to be learned from disability justice.

Critically, the models presented above should not be understood as mutually exclusive. In moving past pathologized ethical frameworks of GAC in youth, a simplistic and definitive approach, while tempting, is unlikely to suffice. Lessons from desire-centred frameworks, informed consent models, and disability justice ought to be used in confluence, so as to foster the most supportive, inclusive, and responsible ethics of care with trans youth. Through this work we can not only support trans youth in embodying self-determination but can also begin the process of undermining settler colonial structures and practices rooted in cisheteropatriarchy that remain deeply entrenched within healthcare institutions.

## Conclusion

### Implications, Limitations & Future Directions

Further work must be done to explore the models discussed in this paper, both theoretically and empirically. The field of GAC, particularly for youth, is new and growing, and the lack of long-term data continues to be a hindrance to clinicians and advocates in supporting their patients and communities. Moreover, this paper was unable to address several key elements of trans medicine. The pathological model has not only historically been used to ethically justify GAC, but also to advocate for insurance coverage. How alternative models can be instrumentalized for the same purposes remains to be seen. This is a critical limitation of the present work, as financial access to GAC is not only imperative but also affects trans youth made most vulnerable through capitalism and classism. Yet, while this was not explicitly explored in our work, much of the shift from the need to treat an illness to the responsibility to empower a choice can be applied to funding as well. More fundamentally, the authors support a critical interrogation of insurance coverage as the model of economics in healthcare in and of itself.

With regard to pediatric ethics, many ethical questions remain, such as approaches to dissenting parents, fertility preservation, and supportive discontinuation of GAC. The present paper also failed to address the vital intersections between trans medicine, race, and racism. While we address the central role of settler colonialism in the creation of gender as pathology, we have not directly addressed the multiple axes of marginalization and unique access needs facing Two Spirit Indigenous youth, as well as trans youth of colour, particularly Black trans youth. Further work must be done to understand these nuanced interactions and best support youth in these situations.^99^ Other intersectional approaches, such as in GAC for disabled youth, require analysis as well, especially due to the frequent removal of autonomy for these communities.

The implications of this paper can expand beyond trans medicine as well. The ethical quandary presented by youth access to GAC offers a more expansive enlightenment on status quo frameworks of pediatric consent. There exists a considerable body of ethical literature critiquing dominant approaches to minors in healthcare in that they severely underestimate youth capacity to consent.^100^ As Alderson notes, adults are typically presumed competent unless they show discernable signs of incompetence; the inverse is true for minors.^101^ In rethinking their approach to trans patients, we ask clinicians to consider expanding this reformulation to their adolescent practice as a whole. Questions of competence, desistence, and rationality are not uncommon in pediatrics, and the frameworks reviewed in this paper may be helpful in many clinical scenarios.

This paper reviews extensive critique of the traditional approaches to GAC provision for trans youth. A framework that justifies such care by pathologizing trans youth and lamenting the tragic outcomes of non-treatment, such as suicide and mental illness, is found to be rooted in cisheteropatriarchy, a central feature of settler colonialism. The landslide of legislation attacking the autonomy of trans youth is a continuation of an ongoing process of colonial attempts to control and oppress “deviance,” in service of white cisheteropatriarchal supremacy.

### Summary

This paper reviews extensive critique of the traditional approaches to GAC provision for trans youth. A framework that justifies such care by pathologizing trans youth and lamenting the tragic outcomes of non-treatment, such as suicide and mental illness, is found to be rooted in cisheteropatriarchy, a central feature of settler colonialism. The landslide of legislation attacking the autonomy of trans youth is a continuation of an ongoing process of colonial attempts to control and oppress “deviance,” in service of white cisheteropatriarchal supremacy. We have also begun a cursory exploration of possible ways forward in the provision of GAC, drawing from Tuck’s desire-centred research, Ashley’s ethics of exploration, applications of the informed consent model, and work in disability justice.

Building a responsible ethical approach to GAC for trans youth is a daunting and delicate task. It is no surprise that well-meaning advocates have relied on the ethical shortcut of pointing to tragedy and illness in order to justify such care, especially in the face of the unconscionable legislative attacks on trans youth we have seen in the past years. Yet, the continued attempts to remove trans youth autonomy and invalidate their personhood make a depathologized ethical approach all the more imperative. We must learn to “embrace discomfort [and] appreciate ethical complexity.”^102^ Diagnostic frameworks of evaluating youth access to GAC participate in the ongoing medical colonization of gender deviance, leading to the erasure of non-binary genders, the requirement of false gender performance, and invasive evaluations that ultimately form a barrier to access. The cumulative impact of these patient-level consequences results in ongoing settler colonial exploitation and expropriation.

Rather than taking this problematic ethical shortcut, we implore clinician advocates to take the long way around. This fundamental switch in the practice of GAC, which challenges rather than reinforces cisheteropatriarchy, leads to improved access to anti-oppressive healthcare, with the potential to uplift rather than undermine broader anti-colonial movements towards liberation. We must do the uncomfortable work of rethinking GAC for trans youth, as well as trans medicine more broadly. Only through this discomfort can a truly liberatory framework of GAC for trans youth be achieved.

## Note

The authors have no conflicts to disclose.
